# Tooth Loss and Head and Neck Cancer: A Meta-Analysis of Observational Studies

**DOI:** 10.1371/journal.pone.0079074

**Published:** 2013-11-15

**Authors:** Xian-Tao Zeng, Wei Luo, Wei Huang, Quan Wang, Yi Guo, Wei-Dong Leng

**Affiliations:** 1 Department of Stomatology, Taihe Hospital, Hubei University of Medicine, Shiyan, People’s Republic of China; 2 Institute of Stomatology, General Hospital of Chinese People’s Liberation Army, Beijing, People’s Republic of China; 3 Department of Epidemilogy, School of Public Health, Wuhan University, Wuhan, People’s Republic of China; Virginia Commenwealth University, United States of America

## Abstract

**Backgroud:**

Epidemiological studies have shown that tooth loss is associated with risk of head and neck cancer (HNC); however, the results were inconsistent. Therefore, we conducted a meta-analysis to ascertain the relationship between tooth loss and HNC.

**Methods:**

We searched for relevant observational studies that tested the association between tooth loss and risk of HNC from PubMed and were conducted up to January 30, 2013. Data from the eligible studies were independently extracted by two authors. The meta-analysis was performed using the Comprehensive Meta-Analysis 2.2 software. Sensitivity and subgroup analyses were conducted to evaluate the influence of various inclusions. Publication bias was also detected.

**Results:**

Ten articles involving one cohort and ten case-control studies were yielded. Based on random-effects meta-analysis, an association between tooth loss and HNC risk was identified [increased risk of 29% for 1 to 6 teeth loss (OR = 1.29, 95% CI = 0.52–3.20, p = 0.59), 58% for 6 to 15 teeth loss (OR = 1.58, 95% CI = 1.08–2.32, p = 0.02), 63% for 11+ teeth loss (OR = 1.63, 95% CI = 1.23–2.14, p<0.001), 72% for 15+ teeth loss (OR = 1.72, 95% CI = 1.26–2.36, p<0.001), and 89% for 20+ teeth loss (OR = 1.89, 95% CI = 1.27–2.80, p<0.001)]. The sensitivity analysis shows that the result was robust, and publication bias was not detected.

**Conclusions:**

Based on the current evidence, tooth loss is probably a significant and dependent risk factor of HNC, which may have a dose-response effect. People who lost six or more teeth should pay attention to symptoms of HNC, and losing 11 teeth or 15 teeth may be the threshold.

## Introduction

Head and neck cancer (HNC) mainly originates in the oral cavity, pharynx, and larynx. HNC accounts for 12% of all malignancies worldwide. An estimated total of 400000 cases of oral cavity and pharynx diseases, 160000 cases of laryngeal cancer, and 300000 mortality per year [1]. Therefore, finding and preventing the risk factors are important and significant research areas. In the past decades, smoking (active and passive), alcohol, genetic factors, viral infection (mostly human papillomavirus), sex, and occupational exposure have been identified as significant risk factors for HNC. Among these factors, smoking and alcohol are the most significant [2], [3]. Tooth loss has been considered to influence food choice, diets, nutrition intake, and esthetics significantly [4]. A systematic review and meta-analysis provided that tooth loss is associated with the impairment of the oral health-related quality of life and the location and distribution of tooth loss significantly and independently affect the severity of the impairment [5]. Epidemiological studies has shown that age, gender, diabetes, social and geographical disparities, smoking, patients and dentists attitudes on oral health status, and alcohol are the risk factors of tooth loss [6], [7], [8], [9].

Both HNC and tooth loss share common risk factors; moreover, given their special anatomic location, an interesting assumption was formed on whether or not an association between tooth loss and HNC existed? Zheng et al (1990) [10] first investigated the association between HNC and tooth loss, and found that tooth loss is a strong risk factor for oral cancer in both males and females. Since then, many relevant studies have been published. However, these studies provided inconsistent or even contradictory results. In addition, the threshold on the number of missing tooth that both patients and dentists showed pay attention to remains unclear. Therefore, we performed this meta-analysis according to the Preferred Reporting Items for Systematic Reviews and Meta-Analyses (PRISMA) statement (File S1) [11] and Meta-analysis of Observational Studies in Epidemiology (MOOSE) guidelines [12], to obtain a more precise estimation on the association between tooth loss and HNC.

## Methods

### Eligibility Criteria

Cohort, case-control, and cross-sectional studies that evaluated the association between tooth loss and HNC and those that meet the following criteria were considered eligible for inclusion: (1) full-text could be obtained; (2) clear diagnostic criteria for HNC and definition of tooth loss were reported; and (3) the adjusted and/or unadjusted hazard ratios (HRs), odds ratios (ORs), or relative risks (RRs), and the associated 95% confidence intervals (CIs), or the numbers of events that can calculate these factors, were reported. If more than one study covered the same population, only the report containing the most comprehensive information on that population was included. Two authors independently evaluated the eligibility of all the retrieved studies, and disagreements were resolved by discussion.

### Search Strategy

The PubMed database was searched up to January 30, 2013 (re-searched on August 31, 2013) for published studies that tested the association between tooth loss and HNC. The search term (“head and neck cancer” OR “oral cancer” OR “oropharyngeal cancer” OR “pharyngeal cancer” OR “laryngeal cancer”) AND (“dentition” OR “tooth loss”) was used. We also reviewed the reference lists of included articles and recent reviews.

### Data Extraction

Two authors independently collected and tabulated the following information of each eligible study: the first author’s surname, year of publication, study design, country of origin, sample size, number of events, age range, assessment of tooth loss and HNC, tumor site and pathologic type of HNC, crude or adjusted point estimates and relevant 95% CIs, and the covariates for the adjusted point estimates.

The design of most of the included studies was a case-control study and reported ORs. Only one study was prospective cohort and reported HR [13]. We directly considered HR as RR, and then transformed RR into OR by using the following formula [14]: 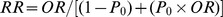, where P_0_ is the incidence of the outcome of interest in the non-exposed group. The standard error (SE) of the resulting converted OR was then determined using the following formula: 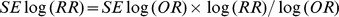. Given that these transformations can overestimate the variance of OR derived from RR [15], we performed a sensitivity analysis by omitting the study.

The numbers of lost tooth varied in the included studies; hence, we gathered and categorized these teeth into five categories as follows: lost 1 to 6 teeth, 6 to 15 teeth, 11^+^ teeth, 15^+^ teeth, and 20^+^ teeth.

### Data Analysis

We computed a pooled OR and relevant 95% CI by using the Comprehensive Meta-Analysis software, version 2.2 (Biostat, Englewood, New Jersey) [16] to generate the forest plots and to assess heterogeneity of the included studies. Heterogeneity was quantified using the Q and I^2^ statistics [17], and the heterogeneity was defined as low, moderate, and high based on I^2^ values of 25%, 50%, and 75%, respectively [18]. When the I^2^≤25%, which indicates no evidence of heterogeneity, we used the fixed-effect model; otherwise, we used the random-effects model. In the presence of heterogeneity, we performed sensitivity analysis to explore the possible explanations for heterogeneity by removing each study in each turn to test the robustness of the main results or by switching the fixed and random effects models.

We used the Stata 12.0 software for the dose-response estimates based on pooled ORs and 95% CIs by each category of the number of lost teeth. Publication bias was assessed by visual inspection of the funnel plots and the Egger linear regression test [19]. In addition, we calculated the number of unpublished studies that would negate the results and the pooled OR adjusted for publication bias by using the ‘trim and fill’ method to assess the effect of possible publication bias [20].

## Results

### Study Selection and Characteristics

**Figure 1:**
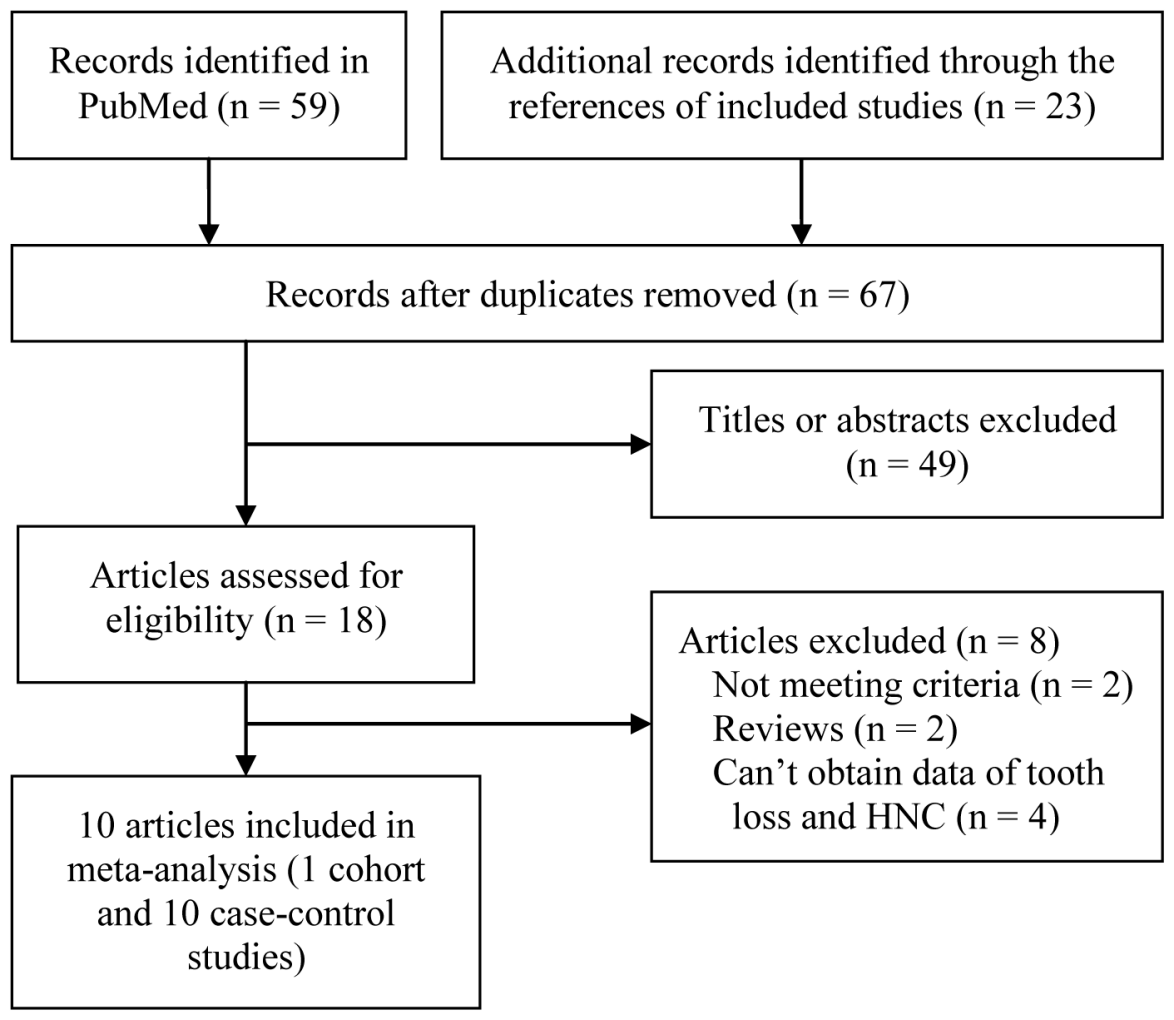
Flow chart from identification of eligible studies to final inclusion. HNC, head and neck cancer.

From the 82 records initially found, 10 articles involving 11 case-control studies [10], [21], [22], [23], [24], [25], [26], [27], [28], [29] and one cohort study [13] were included in this meta-analysis. A detailed flow chart of the selection process is shown in Fig. 1.

From the included studies, the study of Guha et al. [27] contains two multicentric case-control studies from central Europe (including Romania, Poland, and Russia) and Latin America (including Cuba, Argentina, and Brazil), wheras the other 10 were single center studies. All of the cases were histologically, pathologically or cytologically confirmed as HNC, and clearly defined the referenced group of tooth loss, with the major characteristics presented in Table 1. All of the studies reported adjusted the point estimates and 95% CIs. The adjusted covariates are shown in Table 2.

**Table 1:** Characteristics of included studies in the meta-analysis.

References	Country	Study design	Sample sizes(case/control)	Age (yrs)	Outcomes	Definition of reference group
Zheng 1990	China	Case-control	404/404	18 to 80	Oral cavity and pharynx	No lost tooth
Marshall 1992	USA	Case-control	290/290	<50 to 76+	Oral cavity and pharynx	No lost tooth
Bundgaard 1995	Denmark	Case-control	161/400	≤45 to >75	Oral cavity and pharynx	Number of 15+ teeth
Talamini 2000	Italy	Case-control	132/148	27 to 86	Oral cavity and pharynx	Lost ≤5 teeth
Garrote 2001	Cuba	Case-control	200/200	<55 to ≥75	Oral cavity and pharynx	Lost ≤5 teeth
Lissowska 2003	Poland	Case-control	122/124	<45 to ≥75	Oral cavity and pharynx	Lost 0 to5 teeth
Rosenquist 2005	Sweden	Case-control	132/320	33 to 89	OOSCC	No lost tooth
Guha E 2007	Central Europe	Case-control	792/928	All ages	HNSCC	Lost ≤5 teeth
Guha LA 2007	Latin America	Case-control	2113/1805	All ages	HNSCC	Lost ≤5 teeth
Hiraki 2008	Japanese	Case-control	429/858	20 to 79	Head an neck	Number of ≥21 teeth
Michaud 2008	USA	Cohort	118	40 to 75	Oropharyngeal	Number of 23 to32 teeth
Divaris 2010	USA	Case-control	1389/1396	20 to 80	HNSCC	Lost 0 to 5 teeth

OOSCC, oral and oropharyngeal squamous cell carcinoma; HNSCC, head and neck squamous cell carcinoma; Guha E 2007, the study conducted in Europe; Guha LA 2007, the study conducted in Latin-America.

**Table 2:** Adjustments in studies included in this meta-analysis.

References	Adjustment
Zheng 1990	age, gender, tobacco, alcohol, and education
Marshall 1992	tobacco and alcohol
Bundgaard 1995	tobacco and alcohol
Talamini 2000	age, gender, tobacco, alcohol, and fruit and vegetable intake
Garrote 2001	gender, age, area of residence, education, tobacco, and alcohol
Lissowska 2003	tooth brushing
Rosenquist 2005	tobacco and alcohol
Guha 2007	age, gender, country, education, tobacco, alcoho, and all other oral health variables
Hiraki 2008	age, gender, tobacco, alcohol, vegetable and fruit intake, body mass index, and regular exercise
Michaud 2008	age, race, physical activity, history of diabetes, alcohol, body-mass index, geographical location, height, calcium intake, total calorific intake, red-meat intake, fruit and vegetable intake, vitamin D score, and tobacco
Divaris 2010	age, gender, race, education, tobacco, alcohol, and fruit and vegetable consumption

### Tooth Loss and Risk of HNC

**Figure 2:**
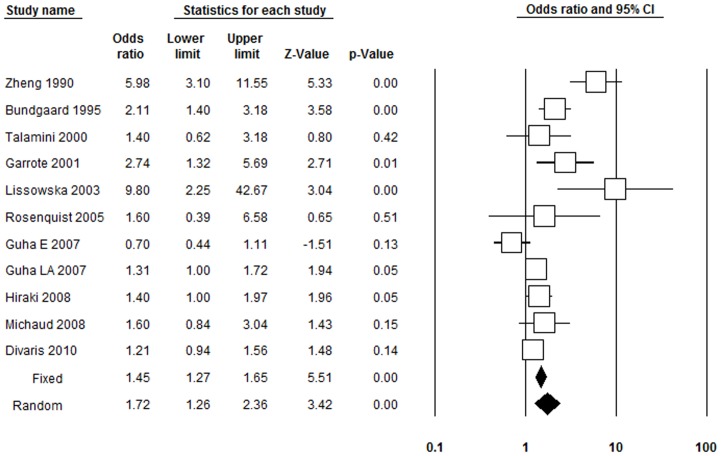
Forest plot of 15^+^ teeth loss and risk of head and neck cancer, studies are pooled with random-effects model. Guha E 2007, the study conducted in Europe; Guha LA 2007, the study conducted in Latin-America.

Three studies reported 1 to 6 teeth loss and risk of HNC. Among the three studies, one [10] showed a significantly positive association between tooth loss and the risk of HNC, the other two were negative [21], [26]. Overall, no association between 1 to 6 teeth loss and HNC (OR = 1.29, 95% CI = 0.52–3.20, p = 0.59; Table 3) was observed. Substantial heterogeneity was observed (p<0.001, I^2^ = 85.59%). Nine articles involving 10 studies [10], [13], [21], [23], [24], [25], [26], [27], [28] reported 6 to 15 teeth loss and risk of HNC, where obviously heterogeneity was observed (p<0.001, I^2^ = 82.92%). The result from the random-effects model showed that 6 to 15 teeth loss could significantly increase the risk of developing HNC by 1.58 times (OR = 1.58, 95% CI = 1.08–2.32, p = 0.02; Table 3). All the included studies reported their result based on the random-effects model (p<0.001, I^2^ = 74.41%) of 11^+^ teeth loss and the risk of HNC. A significantly increased risk for developing HNC by 1.63 times was observed (OR = 1.63, 95% CI = 1.23–2.14, p<0.001; Table 3). All of the included studies reported 15^+^ teeth loss and the risk of HNC. The result of the meta-analysis showed that 15^+^ teeth loss could significantly increase the risk of HNC by 1.72 times (OR = 1.72, 95% CI = 1.26–2.36, p<0.001; Fig. 2). Substantial heterogeneity was observed (p<0.001, I^2^ = 76.53%). The pooled result of the three case-control studies [22], [26], [29] indicated that exposure to 20^+^ teeth loss could increase the risk of HNC by 1.89 times (OR = 1.89, 95% CI = 1.27–2.80, p<0.001; Table 3) based on the random-effects model (p = 0.14, I^2^ = 49.93%).

**Table 3:** Results of overall and subgroups analyses of pooled ORs and 95% Cis.

Total and subgroups	1 to 6 teeth loss			6 to 15 teeth loss			11+ teeth loss			15+ teeth loss			20+ teeth loss		
	N	OR(95% CI)	I2(%)	N	OR(95% CI)	I2(%)	N	OR(95% CI)	I2(%)	N	OR(95% CI)	I2(%)	N	OR(95% CI)	I2(%)
**Total (REM)**	3	1.29(0.52–3.20)	85.59	10	1.58(1.08–2.32)	82.92	11	1.63(1.23–2.14)	74.41	11	1.72(1.26–2.36)	76.53	3	1.89(1.27–2.80)	49.93
**Total (FEM)**	3	0.89(0.71–1.11)	85.59	10	1.37(1.19–1.58)	82.92	11	1.39(1.24–1.57)	74.41	11	1.45(1.27–1.65)	76.53	3	1.75(1.35–2.27)	49.93
**Study design**															
Cohort	0	NA	NA	1	1.18(0.69–1.65)	NA	1	1.60(0.84–3.04)	NA	1	1.60(0.84–3.04)	NA	NA	NA	NA
Case-control	0	NA	NA	9	1.65(1.08–2.52)	84.72	10	1.63(1.22–2.60)	76.86	10	1.75(1.25–2.45)	78.83	NA	NA	NA
**Definition of reference group**															
No lost tooth	3	1.29(0.52–3.20)	85.59	3	2.05(0.56–7.43)	93.12	3	3.49(1.66–7.34)	47.14	2	3.61(1.03–12.68)	63.59	1	2.40(1.40–4.10)	NA
Number of teeth	0	NA	NA	6	1.27(0.97–1.68)	49.21	7	1.34(1.06–1.69)	64.06	8	1.48(1.10–1.97)	70.51	2	1.72(1.03–2.87)	55.73
**Country origin**															
Asia	1	3.99(1.75–9.08)	NA	1	6.53(3.98–10.71)	0	2	2.66(0.58–12.21)	94.83	2	2.81(0.68–11.64)	93.22	1	1.40(1.00–1.97)	NA
USA	1	0.80(0.38–1.70)	NA	3	1.12(0.89–1.42)	0	3	1.47(0.99–2.18)	38.19	2	1.26(0.99–1.59)	0	0	NA	NA
Latin-America	0	NA	NA	2	1.32(1.03–1.68)	0	2	1.75(0.86–3.54)	70.99	2	1.75(0.86–3.54)	70.96	0	NA	NA
Europe	1	0.78(0.61–1.00)	NA	4	1.34(0.74–2.44)	66.24	4	1.31(0.68–2.54)	75.75	5	1.71(0.83–3.52)	79.21	2	2.40(1.60–3.59)	0

REM, random-effects model; FEM, fixed effect model; N, number of trials; OR, odds ratio; CI, confidence interval; NA, not applicable.

**Figure 3:**
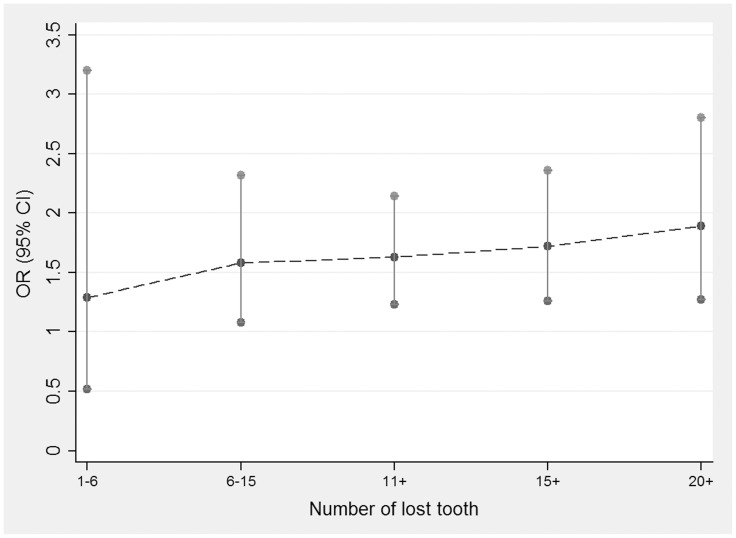
The plot of trend of simulative dose-response effect based on the ORs and corresponding CIs of the numbers of lost teeth and risk of head and neck cancer.

Fig. 3 shows the trend of the simulative dose-response effect based on the ORs and the corresponding CIs of the numbers of lost teeth, which indicated that the association between tooth loss and HNC risk may have a dose-response relationship.

### Sensitivity and Subgroups Analyses

**Figure 4:**
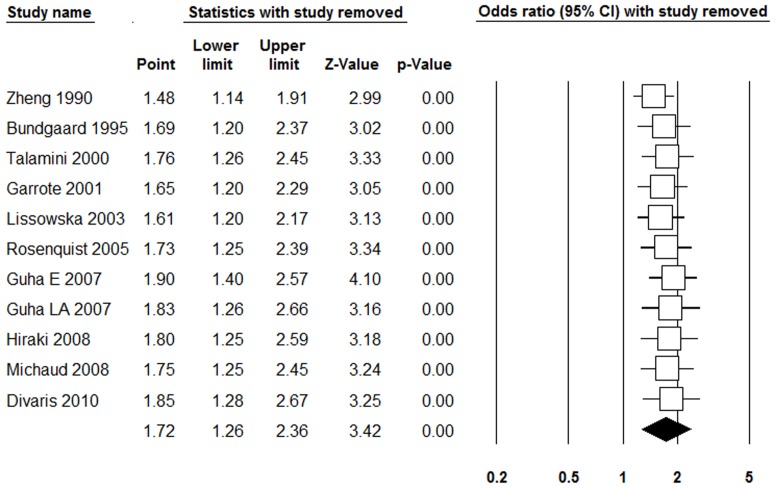
Forst plot of sensitivity analysis by removing each study in each turn for 15^+^ teeth loss and risk of head and neck cancer.

Table 3 shows the results of the sensitivity and subgroups analyses. We switched all the random-effects models to the fixed effect models, which indicated that all the results were not substantial changed. We removed the study of Michaud et al. [13], which reported HR yielded similar results and with substantial evidence of heterogeneity. Further exclusion of any single study did not materially alter the combined OR, with a range from 1.40 (95% CI = 1.14–1.73, p<0.001) to 1.77 (95% CI = 1.35–2.32, p<0.001) of 11^+^ teeth loss, and from 1.48 (95% CI = 1.14–1.91, p<0.001) to 1.90 (95% CI = 1.40–2.57, p<0.001) of 15^+^ teeth loss (Fig. 4). The results of the subgroup analyses were varied, especially for the country of origin.

### Publication Bias

**Figure 5:**
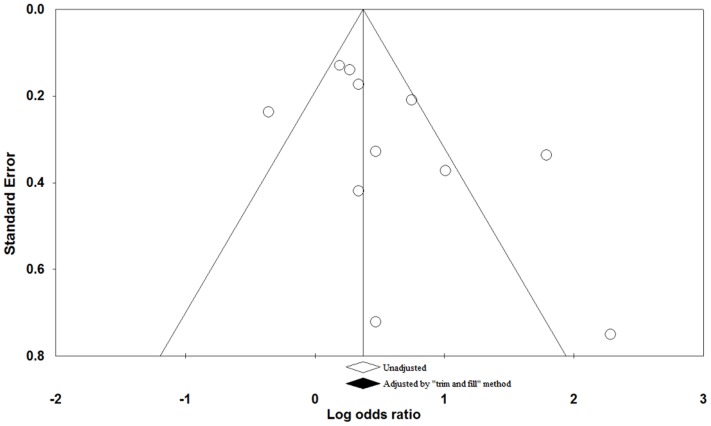
Filled funnel plot with pseudo-95% CIs of results of 11 studies based on the result of 15^+^ teeth loss and risk of head and neck cancer.

Visual inspection of the funnel plot did not identify any substantial asymmetry (Fig. 5) and the Egger linear regression test also indicated no evidence of publication bias among the studies (for 6–15 teeth loss, p = 0.31; for 11+ teeth loss, p = 0.14; for 15+ teeth loss, p = 0.10). The “trim and fill” method identified any possible missing studies (Fig. 5) and the adjustment estimated OR was similar to the original estimate (OR = 1.72, 95% CI = 1.26–2.36).

## Discussion

### Main Findings

The association between tooth loss and HNC is still not fully understood. Our meta-analysis of the 11 case-control studies and one cohort study provides evidence that individuals would face increased risk of HNC by 29% for those with 1 to 6 teeth loss, 58% for 6 to 15 teeth loss, 63% for 11+ teeth loss, 72% for 15+ teeth loss, and 89% for 20+ teeth loss compared with the reference group. These results indicate that greater teeth loss is associated with an increased risk of HNC. In other words, a dose-response relationship exists between tooth loss and HNC (Fig. 3). Except for 1 to 6 teeth loss, the results all have significant statistical difference, which suggests that tooth loss is probably a significant risk factor for HNC.

Tobacco smoking and alcohol drinking are known risk factors for HNC. In our meta-analysis, all the studies adjusted smoking and alcohol, except for one case-control study published in 2003 by Lissowska et al. [25]. Several studies also adjusted age, gender, ethnicity, body mass index, education, and other oral health variables, which suggests that tooth loss is probably an independent risk factor of HNC.

All the included studies contained both males and females. The study by Zheng et al. [10] provided the respective data for males and females, and their results show that females were more vulnerable than males. Thus, regardless if the patient is male or female, tooth loss is probably a risk factor for HNC.

### Sources of Heterogeneity

Substantial heterogeneity was observed among the studies of tooth loss and HNC risk, which is not surprising because of the differences in the characteristics of populations, definition of the reference and tooth loss group(s), and the adjustment for confounding factors. Our sensitivity analysis (by changing the effect models, removing the cohort study, and omitting every single study each time) and subgroup analysis (by study design, definition of reference group, and country origin) results provide evidence that country of origin and definition of reference group probably contributed to the heterogeneity.

### Strengths and Limitations

The major strength of our study is that this it is the first to perform meta-analysis on this topic. We searched relevant published studies via electronic and hand searching. To the best of our knowledge, we have collected all published studies that met the inclusion criteria and the publication bias test also provided no evidence of publication bias. Moreover, we performed sensitivity analysis by using three methods and subgroups analysis based on the study design, definition of the reference group, country of origin, which could improve the reliability of the results and reduce the performance bias of the meta-analysis. Third, the association of tooth loss with the risk of HNC persisted and remained without substantial change in the sensitivity analyses based on various methods. In addition, the subgroup analysis results indicate that, with accumulating evidence and enlarged sample sizes, the statistical power is enhanced to provide more precise and reliable risk estimates. Finally, our results were based on adjusted estimates, thereby making the result more credible than unadjusted ones.

However, there was an interesting finding when we re-searched the PubMed database on August 31, 2013. We found there was a similar paper by Wang RS et al [30] have been published in PLoS One on Aug 29, 2013 (http://www.ncbi.nlm.nih.gov/pubmed/23990929). Their work is perfect! However, when we compared our meta-analysis to theirs, we found there were three major differences/advantages of our meta-analysis. First, the deadline of search of theirs was March, 2013 and yielded eight case-control studies and one cross-sectional study; however, the deadline of search in our meta-analysis was January 30, 2013 and yielded 10 articles involving 11 case-control studies and one cohort study. Obviously, our search is more comprehensive. Second, we performed meta-analysis based on the number of lost tooth (Table 3); however, they pooled all studies together and ignored the influence of difference number lost tooth, which may biased the results. Third, our meta-analysis conducted a simulative dose-response effect analysis based on the number of lost tooth; this may provide more reference information than their meta-analysis.

However, our study also has some limitations. First, the definition of the reference group and tooth loss used among studies varied, and the former might be the source of heterogeneity. No international unification index of evaluating tooth loss for relevant studies is available, which caused heterogeneity, and increased the difficulty performing the meta-analysis, and even resulted in the failure of the criteria for the meta-analysis. Second, heterogeneity was detected. Although heterogeneity between studies is very common in the meta-analysis of observational studies, we did not ignore it. We performed subgroup analyses to verify the heterogeneity, but it was still observed. Third, we were unable to investigate the histopathological subtypes because only 3 studies [26], [27], [28] clearly reported that the type of cancer is squamous cell carcinoma, wheras others are mixed. Finally, the results are significantly inconsistent based on the subgroups analyses and the statistical power was limited because of a relatively small number of major included studies. Four, the examination of tooth loss and HNC has not been the primary association of interest for many studies, and as a result, adjustment for other ‘proximal’ variables such as caries, periodontitis, alcoholic mouthwash use, or reason for extractions/tooth loss is rarely/never done. Therefore, the facticity of results might be influenced by this.

### Implications for Further Research

Based on the results our meta-analysis, several questions arise. First, in routine clinical work, we found the oral health of patients who undergo HNC are more severe; hence, the association of tooth loss with HNC might or might not be causal. A prospective cohort study design with enough follow-up time and adequate control for confounding factors is needed to answer this question. Second, tooth loss is not acceptable and can potentially influence future demand for treatment [31]. Thus, patients should seek treatment if they lose their teeth. Obviously, individuals in developed countries can obtain more convenient and better oral healthcare. Therefore, is there a difference between developed and developing countries because of the social economic differences? Thus, studies should perform stratified analysis based on social economics and should respectively report the results. Third, what are the exact mechanisms in which tooth loss independently increase the risk of HNC? To answer this question, we suggest that experimental studies be conducted. Fourth, is there a dose-response effect between tooth loss and HNC risk? Although our study indicated that dose-response effects exist, the numbers of lost teeth overlapped with one another. Therefore, further studies should answer this question with sequential or without repeated numbers, and they should explore the critical value numbers. Finally, could preventing or treating tooth loss decrease the risk of HNC? Well-designed clinical trials, especially randomized controlled trials, are suggested to answer this question. Finally, we suggest further relevant studies can take tooth loss as the primary interesting.

### Conclusions

This meta-analysis indicates that tooth loss is probably a significant and dependent risk factor of HNC, which may have a dose-response effect. People who lost six or more teeth should pay attention to symptoms of HNC, and losing 11 teeth or 15 teeth may be the threshold.

## Supporting Information

Checklist S1 **PRISMA Checklist.**
(DOC)

## References

[1] Wozniak A, Szyfter K, Szyfter W, Florek E (2012) [Head and neck cancer–history]. Przegl Lek 69: 1079–1083. 23421095

[2] Conway DI, Hashibe M, Boffetta P, consortium I, Wunsch-Filho V, et al (2009) Enhancing epidemiologic research on head and neck cancer: INHANCE - The international head and neck cancer epidemiology consortium. Oral Oncol 45: 743–746. 19442571 10.1016/j.oraloncology.2009.02.007

[3] Mehanna H, Paleri V, West CM, Nutting C (2010) Head and neck cancer–Part 1: Epidemiology, presentation, and prevention. BMJ 341: c4684. 20855405 10.1136/bmj.c4684

[4] Adegboye AR, Twetman S, Christensen LB, Heitmann BL (2012) Intake of dairy calcium and tooth loss among adult Danish men and women. Nutrition 28: 779–784. 22459555 10.1016/j.nut.2011.11.011

[5] Gerritsen AE, Allen PF, Witter DJ, Bronkhorst EM, Creugers NH (2010) Tooth loss and oral health-related quality of life: a systematic review and meta-analysis. Health Qual Life Outcomes 8: 126. 21050499 10.1186/1477-7525-8-126 PMC2992503

[6] Taylor GW, Manz MC, Borgnakke WS (2004) Diabetes, periodontal diseases, dental caries, and tooth loss: a review of the literature. Compend Contin Educ Dent 25: 179–184, 186–178, 190; quiz 192. 15641324

[7] Matthews JC, You Z, Wadley VG, Cushman M, Howard G (2011) The association between self-reported tooth loss and cognitive function in the REasons for Geographic And Racial Differences in Stroke study: an assessment of potential pathways. J Am Dent Assoc 142: 379–390. 21454843 10.14219/jada.archive.2011.0192 PMC3744362

[8] Anand PS, Kamath KP, Shekar BR, Anil S (2012) Relationship of smoking and smokeless tobacco use to tooth loss in a central Indian population. Oral Health Prev Dent 10: 243–252. 23094267

[9] Heegaard K, Avlund K, Holm-Pedersen P, Hvidtfeldt UA, Bardow A, et al (2011) Amount and type of alcohol consumption and missing teeth among community-dwelling older adults: findings from the Copenhagen Oral Health Senior study. J Public Health Dent 71: 318–326. 22320290 10.1111/j.1752-7325.2011.00276.x

[10] Zheng TZ, Boyle P, Hu HF, Duan J, Jian PJ, et al (1990) Dentition, oral hygiene, and risk of oral cancer: a case-control study in Beijing, People’s Republic of China. Cancer Causes Control 1: 235–241. 2102296 10.1007/BF00117475

[11] Moher D, Liberati A, Tetzlaff J, Altman DG (2009) Group P (2009) Preferred reporting items for systematic reviews and meta-analyses: the PRISMA statement. BMJ 339: b2535. 19622551 10.1136/bmj.b2535 PMC2714657

[12] Stroup DF, Berlin JA, Morton SC, Olkin I, Williamson GD, et al (2000) Meta-analysis of observational studies in epidemiology: a proposal for reporting. Meta-analysis Of Observational Studies in Epidemiology (MOOSE) group. JAMA 283: 2008–2012. 10789670 10.1001/jama.283.15.2008

[13] Michaud DS, Liu Y, Meyer M, Giovannucci E, Joshipura K (2008) Periodontal disease, tooth loss, and cancer risk in male health professionals: a prospective cohort study. Lancet Oncol 9: 550–558. 18462995 10.1016/S1470-2045(08)70106-2 PMC2601530

[14] Zhang J, Yu KF (1998) What’s the relative risk? A method of correcting the odds ratio in cohort studies of common outcomes. JAMA 280: 1690–1691. 9832001 10.1001/jama.280.19.1690

[15] Greenland S (2004) Model-based estimation of relative risks and other epidemiologic measures in studies of common outcomes and in case-control studies. Am J Epidemiol 160: 301–305. 15286014 10.1093/aje/kwh221

[16] Borenstein M, Hedges L, Rothstein H (2005) Comprehensive Meta-analysis. Version 2 ed. Biostat, Englewood, New Jersey.

[17] Higgins JP, Thompson SG (2002) Quantifying heterogeneity in a meta-analysis. Stat Med 21: 1539–1558. 12111919 10.1002/sim.1186

[18] Higgins JP, Thompson SG, Deeks JJ, Altman DG (2003) Measuring inconsistency in meta-analyses. BMJ 327: 557–560. 12958120 10.1136/bmj.327.7414.557 PMC192859

[19] Egger M, Davey Smith G, Schneider M, Minder C (1997) Bias in meta-analysis detected by a simple, graphical test. BMJ 315: 629–634. 9310563 10.1136/bmj.315.7109.629 PMC2127453

[20] Duval S, Tweedie R (2000) Trim and fill: A simple funnel-plot-based method of testing and adjusting for publication bias in meta-analysis. Biometrics 56: 455–463. 10877304 10.1111/j.0006-341x.2000.00455.x

[21] Marshall JR, Graham S, Haughey BP, Shedd D, O’Shea R, et al (1992) Smoking, alcohol, dentition and diet in the epidemiology of oral cancer. Eur J Cancer B Oral Oncol 28B: 9–15. 1422474 10.1016/0964-1955(92)90005-l

[22] Bundgaard T, Wildt J, Frydenberg M, Elbrond O, Nielsen JE (1995) Case-control study of squamous cell cancer of the oral cavity in Denmark. Cancer Causes Control 6: 57–67. 7718736 10.1007/BF00051681

[23] Talamini R, Vaccarella S, Barbone F, Tavani A, La Vecchia C, et al (2000) Oral hygiene, dentition, sexual habits and risk of oral cancer. Br J Cancer 83: 1238–1242. 11027440 10.1054/bjoc.2000.1398 PMC2363583

[24] Garrote LF, Herrero R, Reyes RM, Vaccarella S, Anta JL, et al (2001) Risk factors for cancer of the oral cavity and oro-pharynx in Cuba. Br J Cancer 85: 46–54. 11437401 10.1054/bjoc.2000.1825 PMC2363910

[25] Lissowska J, Pilarska A, Pilarski P, Samolczyk-Wanyura D, Piekarczyk J, et al (2003) Smoking, alcohol, diet, dentition and sexual practices in the epidemiology of oral cancer in Poland. Eur J Cancer Prev 12: 25–33. 12548107 10.1097/00008469-200302000-00005

[26] Rosenquist K, Wennerberg J, Schildt EB, Bladstrom A, Goran Hansson B, et al (2005) Oral status, oral infections and some lifestyle factors as risk factors for oral and oropharyngeal squamous cell carcinoma. A population-based case-control study in southern Sweden. Acta Otolaryngol 125: 1327–1336. 16303683 10.1080/00016480510012273

[27] Guha N, Boffetta P, Wunsch Filho V, Eluf Neto J, Shangina O, et al (2007) Oral health and risk of squamous cell carcinoma of the head and neck and esophagus: results of two multicentric case-control studies. Am J Epidemiol 166: 1159–1173. 17761691 10.1093/aje/kwm193

[28] Divaris K, Olshan AF, Smith J, Bell ME, Weissler MC, et al (2010) Oral health and risk for head and neck squamous cell carcinoma: the Carolina Head and Neck Cancer Study. Cancer Causes Control 21: 567–575. 20049634 10.1007/s10552-009-9486-9 PMC2925153

[29] Hiraki A, Matsuo K, Suzuki T, Kawase T, Tajima K (2008) Teeth loss and risk of cancer at 14 common sites in Japanese. Cancer Epidemiol Biomarkers Prev 17: 1222–1227. 18483345 10.1158/1055-9965.EPI-07-2761

[30] Wang RS, Hu XY, Gu WJ, Hu Z, Wei B (2013) Tooth loss and risk of head and neck cancer: a meta-analysis. PLoS One 8: e71122. 23990929 10.1371/journal.pone.0071122 PMC3747175

[31] Cronin M, Meaney S, Jepson NJ, Allen PF (2009) A qualitative study of trends in patient preferences for the management of the partially dentate state. Gerodontology 26: 137–142. 19490136 10.1111/j.1741-2358.2008.00239.x

